# Supplementary material for: Identification and Characterization of a Phase-Variable Element That Regulates the Autotransporter UpaE in Uropathogenic Escherichia coli
Source: mBio. 2018 Aug 7;9(4):e01360-18. doi: 10.1128/mBio.01360-18 (PMC6083910; doi:10.1128/mBio.01360-18)
Supplement: FIG S1 [file mbo004184000sf1.pdf]

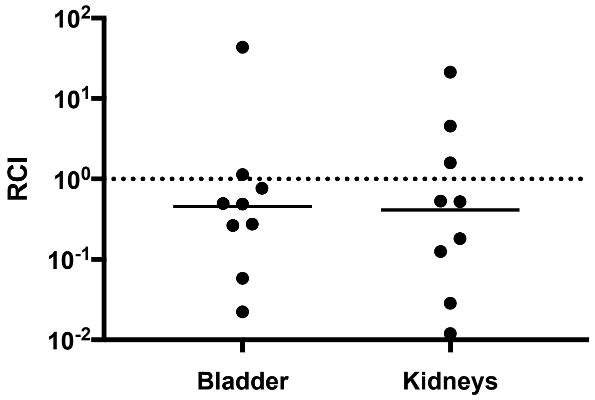

FIG S1. 72HR competitive infection of WAM5048 (CFT073 *ΔipuR/upaE)* vs. WAM4520 (CFT073 *ΔlacZYA*). Relative competitive indices were calculated from bladder and kidney homogenates at 72hpi with lines representing the medians.
